# Learning, Memory, and Executive Function in New MDMA Users: A 2-Year Follow-Up Study

**DOI:** 10.3389/fnins.2015.00445

**Published:** 2015-12-08

**Authors:** Daniel Wagner, Simon Tkotz, Philip Koester, Benjamin Becker, Euphrosyne Gouzoulis-Mayfrank, Joerg Daumann

**Affiliations:** Department of Psychiatry and Psychotherapy, University of CologneCologne, Germany

**Keywords:** MDMA, cognition, verbal memory

## Abstract

3,4-Methylenedioxymethamphetamine (MDMA) is associated with changes in neurocognitive performance. Recent studies in laboratory animals have provided additional support for the neurodegeneration hypothesis. However, results from animal research need to be applied to humans with caution. Moreover, several of the studies that examine MDMA users suffer from methodological shortcomings. Therefore, a prospective cohort study was designed in order to overcome these previous methodological shortcomings and to assess the relationship between the continuing use of MDMA and cognitive performance in incipient MDMA users. It was hypothesized that, depending on the amount of MDMA taken, the continued use of MDMA over a 2-year period would lead to further decreases in cognitive performance, especially in visual paired association learning tasks. Ninety-six subjects were assessed, at the second follow-up assessment: 31 of these were non-users, 55 moderate-users, and 10 heavy-users. Separate repeated measures analyses of variance were conducted for each cognitive domain, including attention and information processing speed, episodic memory, and executive functioning. Furthermore, possible confounders including age, general intelligence, cannabis use, alcohol use, use of other concomitant substances, recent medical treatment, participation in sports, level of nutrition, sleep patterns, and subjective well-being were assessed. The Repeated measures analysis of variance (rANOVA) revealed that a marginally significant change in immediate and delayed recall test performances of visual paired associates learning had taken place within the follow-up period of 2 years. No further deterioration in continuing MDMA-users was observed in the second follow-up period. No significant differences with the other neuropsychological tests were noted. It seems that MDMA use can impair visual paired associates learning in new users. However, the groups differed in their use of concomitant use of illicit drugs. Therefore, performance differences between the groups cannot completely ascribed to the use of MDMA.

## Introduction

3,4-Methylenedioxymethamphetamine (MDMA) is a psychostimulant drug which is commonly referred to as “ecstasy.” MDMA has historically been linked to the electronic dance-music scene, and its use is concentrated among young adults, especially young males. Although, a decline in the popularity of ecstasy was recorded for the years, 2005–2010, the data from 2011 shows that this decrease appears to have been temporary, with a new increase in ecstasy use in some countries^1^. In 2012, the prevalence of ecstasy use in the 15–34-year-old population was 0.6–12.4% in Europe, with the highest prevalence in the United Kingdom (12.4%) and the Netherlands (11.6%)^2^. In North America the prevalence of ecstasy use is even higher than in Europe (U.N.O.o.D.a.C (UNODC), 2013).

A plethora of studies concerning the neurotoxic effects of MDMA in animals and humans supports the neurodegeneration hypothesis with evidence for selective damage to serotonergic axon terminals in the central nervous system (CNS) in laboratory animals, (Parrott, 2013a). Different investigators found impairments in non-spatial, spatial and reference-memory performance tasks in rats after the administration of MDMA (Camarasa et al., 2008; Arias-Cavieres et al., 2010; Kay et al., 2010; McAleer et al., 2013). Interestingly, due to the administration of Memantine, impairments in memory and learning performances due to MDMA seemed to be reversible in rats (Camarasa et al., 2008). However, the findings from animal research can only be applied to humans with caution (Green et al., 2012). Factors like the administration of MDMA, its bioavailability, concentration-dose relationships, possible active metabolites and concentration, as well as dependent plasma protein bindings differ among species and this can affect the pharmacokinetics and pharmacodynamics of MDMA (Green et al., 2012). Nevertheless, extensive empirical evidence exists that shows the negative effects of MDMA on humans, including a reduction in serotonin (5-HT) markers in the CNS (Parrott, 2013a).

The most consistently reported impairments among former and current MDMA users with regard to serotonergic dysfunction include altered executive functioning, as well changes in psychological well-being, neuroendocrine secretion, vegetative functions, the processing of sensory stimuli, sleep architecture, and cognition. However, in most studies deficits in memory were also demonstrated (Parrott, 2013a). Even abstinent MDMA-users seem to have dose-related impairments in delayed visual and immediate verbal memory performances (Bolla et al., 1998). These deficits are linked, among other factors, to the altered serotonergic fiber density in the hippocampus, which is crucial for the processes of learning—and especially vulnerable to the neurotoxic effects of MDMA (Hatzidimitriou et al., 1999; Daumann et al., 2005; Brown et al., 2010; Pergola and Suchan, 2013). Because of the neurotoxic effect of MDMA on the serotonergic fiber density in the hippocampus, many investigations have found deficits in associative learning with poorer immediate and delayed recall abilities in MDMA users compared to non-users (Parrott et al., 1998; Montgomery et al., 2005). Furthermore, those findings were not only demonstrated in heavy MDMA users but also in new MDMA-users after low doses of MDMA use within a short period of lifetime use (Schilt et al., 2007; Wagner et al., 2013).

However, many of the research studies that have examined MDMA users suffered from several methodological problems (e.g., pre-existing differences, polydrug use, differences in lifestyle). In order to account for these shortcomings, we conducted a prospective study. The first results of the current study after a 1-year follow-up period have already been published, (Wagner et al., 2013). In this study, a number of possible interfering variables were explored and taken into account during the analysis of the results (Wagner et al., 2013). Significant effects of immediate and delayed recall of visual paired association learning tasks between MDMA users and controls were found. The authors concluded that MDMA appears to impair visual paired association learning in new users, suggesting serotonergic dysfunction in hippocampal regions as a consequence of MDMA use. In the meantime it has been possible to evaluate the corresponding data from the 2-year follow-up assessment. As with the previous study, the aim of this study was to address the following question: Does the use of MDMA over a period of 2 years lead to changes in cognitive performance with regard to any of the neurocognitive tests?

With respect to the previous findings from the first follow-up assessment, we expect, depending upon the amount of MDMA that was taken, that the continued use of MDMA over a 2-year period will lead to further decreases in cognitive performance, especially in visual paired association learning tasks.

## Methods

### Participants

Hundred and forty-nine new MDMA users with no current physical disorders and no current or previous history of neurological or psychiatric disorders (Axis I and II according to DSM-IV criteria; American Psychiatric Association, 1994) were included in the study. Further exclusion criteria were the following: ingestion of any other illicit psychotropic substances besides cannabis on more than five occasions before the day of the first examination; a history of alcohol misuse (according to DSM-IV criteria, American Psychiatric Association, 1994); and regular medication (except for contraceptives). The main inclusion criterion for the baseline was a high probability of future ecstasy use, operationalized as having some but very limited experience with MDMA (maximum of five pills). The study was carried out between 2006 and 2011 at the department of psychiatry and psychotherapy of the University of Cologne. The participants were invited back after 12 and 24 months. Of the initial 109 subjects present during the second assessment (t1), 96 subjects [63 males, 33 females; age range: 18–41 years, mean age: 22.99 years, standard deviation (*SD*): 4.529] participated in the third assessment (t2). In order to rule out acute intoxication effects, the participants were abstaining from cannabis on both study days during which the cognitive assessment was carried out. It would have been implausible to recruit MDMA users with a longer period of abstinence given the fact that most MDMA users also use cannabis on a regular basis (Parrott et al., 2007). Furthermore, participants had to be abstinent from any other illicit substances for at least seven (7) days in order to rule out acute intoxication effects. Subjects were recruited by placing advertisements in magazines and newspapers and via notifications posted on campus. The study was part of a larger investigation including psychopathological and neuro-imaging measures that will be submitted elsewhere. The study was approved by the Ethics Committee of the Medical Faculty of the University of Cologne in Germany.

### Procedure

All participants were asked to give written informed consent prior to the cognitive assessment. This was followed by a structured interview that, was designed to assess the use of illicit psychotropic substances. For all prevalent psychotropic substances, the interview included questions concerning the participants' age at first use, the number of days since their last use, the average and maximal frequency of their use measured in days per month, their estimated cumulative lifetime dose, their average daily dose, the highest daily dose they had ever used and the duration of their regular use measured in months. For the second and third assessments, the interview covered the following criteria: their age of first use (only assessed if the relevant substance had not been used before), the number of days since their last use, the average and maximal frequency of their use in the last year measured in days per month, their estimated cumulative dose for last year, their average daily dose last year, their highest daily dose last year and the duration of regular use last year measured in months. Qualitative drug screens were performed on the day of the examination by means of urine samples for amphetamines, benzodiazepines, cocaine, methadone, MDMA, and cannabis (enzyme-multiplied immunoassay, von Minden GmbH). Moreover, hair samples were randomly taken in one third of the participants (for financial considerations) and analyzed for the substances, MDMA, MDA, MDEA, amphetamine, methamphetamine, and cannabinoids by the Institute of Legal Medicine at the University of Cologne in order to verify the self-reported substance use. In addition, a questionnaire regarding health behavior was used in order to control for confounding variables such as alcohol and cigarette use, sleep patterns, nutrition, participation in sports, and subjective well-being (*Fragebogen zur Erfassung des Gesundheitsverhaltens; FEG*; (Dlugosch and Krieger, 1995).

### Neuropsychological test battery

The selection of tests in the present study is based on the results of previous cross-sectional and longitudinal studies of MDMA users, which identified alterations in the areas of working memory, learning, memory, and frontal executive functions. For a more detailed description, we refer the reader to our previous study (Wagner et al., 2013). A German version of the Rey Auditory Verbal Learning Test (AVLT) was used to assess verbal declarative memory performance (Heubrock, 1992). The “*Lern- und Gedächtnistest” (LGT 3)* (Bäumler, 1974) is a visual paired association learning task. Figural visual recognition was assessed by a subtest of the *Lern- und Gedächtnistest (LGT; Bäumler,* *1974**)*, which is a classical paired associates learning task. Two subtests of the German version of the Wechsler Intelligence Test (WAIS) were used (Tewes, 1994). The Digit-Span-Test was administered to assess the capacity of the working-memory, and the Digit-Symbol-Test was used to assess the speed of information processing. Moreover, the Stroop Task assessed processing-speed and attention (Stroop, 1935; Bäumler, 1985), and the Trail-making Test measured mental flexibility (Reitan, 1992). We also administered the Raven Standard Progressive Matrices to assess non-verbal general intelligence (Raven et al., 1998).

### Statistical analyses

Three groups of subjects were defined: those who had not used any other illicit drugs apart from cannabis over the course of the 2-year period (non-users); those who had used at least one but not more than 49 ecstasy pills (moderate-users); and those who had used more than 50 pills (heavy-users) over the course of 2 years. In comparison to the first follow-up study, it was possible to assess a group with more heavy MDMA use (see group characteristics). Nevertheless, this sample did not include such heavy use as compared to the other cross-sectional studies mentioned. The group arrangement is based on other studies that had similar or even greater classification ranges with regard to moderate and heavy use (Fox et al., 2001; Reneman et al., 2001)

MDMA users and non-users were compared by means of a one-way analysis of variance according to the following possible confounding variables: age, general intelligence (Raven score), number of days since last cannabis use, duration of regular cannabis use before the initial assessment and duration of regular cannabis use between the second and third assessments. The use of cannabis was measured as the duration of regular use because this has been suggested to have the greatest impact on cognitive performance (Wagner et al., 2010).

In order to assess the participants' health behavior, the authors conducted a one-way analysis of variance of the groups (0 pills, 1–49 pills, 50, or more pills) as independent variables and as dependent variables the computed variables from the questionnaire regarding their health behavior (*Fragebogen zur Erfassung des Gesundheitsverhaltens; FEG*; (Dlugosch and Krieger, 1995). This questionnaire measures different dimensions of the participants' health behavior, including the subjects' satisfaction with their diet, the frequency of their participation in sporting activities (e.g., jogging, bicycling, hiking, walking), their mean consumption of alcoholic drinks (e.g., beer, wine, liquors), their mean drug use (e.g., painkillers, stimulants, tranquilizers, sleeping pills), their sleeping problems and their feelings of subjective well-being.

To evaluate whether the neurotoxic effect of MDMA was affecting the cognitive performance of the participants over the course of 2 years, we conducted three repeated measures analyses of variance (ANOVA) for each separate cognitive domain with the group (0 pills, 1–49 pills, 50 or more pills) as between-subject factor and time of measurement and the cognitive variables as within-subject factors. The first repeated measures ANOVA addressed the subjects' attention and information processing speed (Trail-Making Test part A, Stroop Task parts A and B, digit symbol test). The second repeated measures ANOVA addressed the subjects' episodic memory (AVLT indices, LGT 3 indices). The third repeated measures ANOVA addressed the subjects' frontal/executive functioning (Trail-Making Test part B, Stroop Task part C, digit span test backwards). Furthermore, we computed the effect size of all significant differences, operationally defined as η^2^. All analyses were performed with IBM SPSS statistical software program version 21 (Chicago, IL, USA).

## Results

### Group characteristics

Of the initial 149 subjects who participated in the first assessment, 96 subjects participated in the third assessment after 2 years. Thirty-one subjects did not use any other illicit substance beside cannabis over the course of the 2-year period (non-users). Fifty-five subjects used more than 0.5 but fewer than 49 ecstasy pills (*M* = 10.14; *SD* = 9.98; range: 0.5–45; mean occasion: 1.76; *SD* = 1.45; range: 0–10). Ten subjects used more than 50 ecstasy pills (*M* = 85.80; *SD* = 54.39; range: 51–228; mean occasion: 3.52; *SD* = 1.73; range: 2–6.5). On average the consumption of MDMA declined in both groups in the second follow-up period. We found for moderate-users (t1: *M* = 6.42; *SD* = 7.33; t2: *M* = 3.71; *SD* = 5.3) and for heavy-users (t1: *M* = 51.90; *SD* = 37.28; t2: *M* = 33.9; *SD* = 25.46).

**Figure 1:**
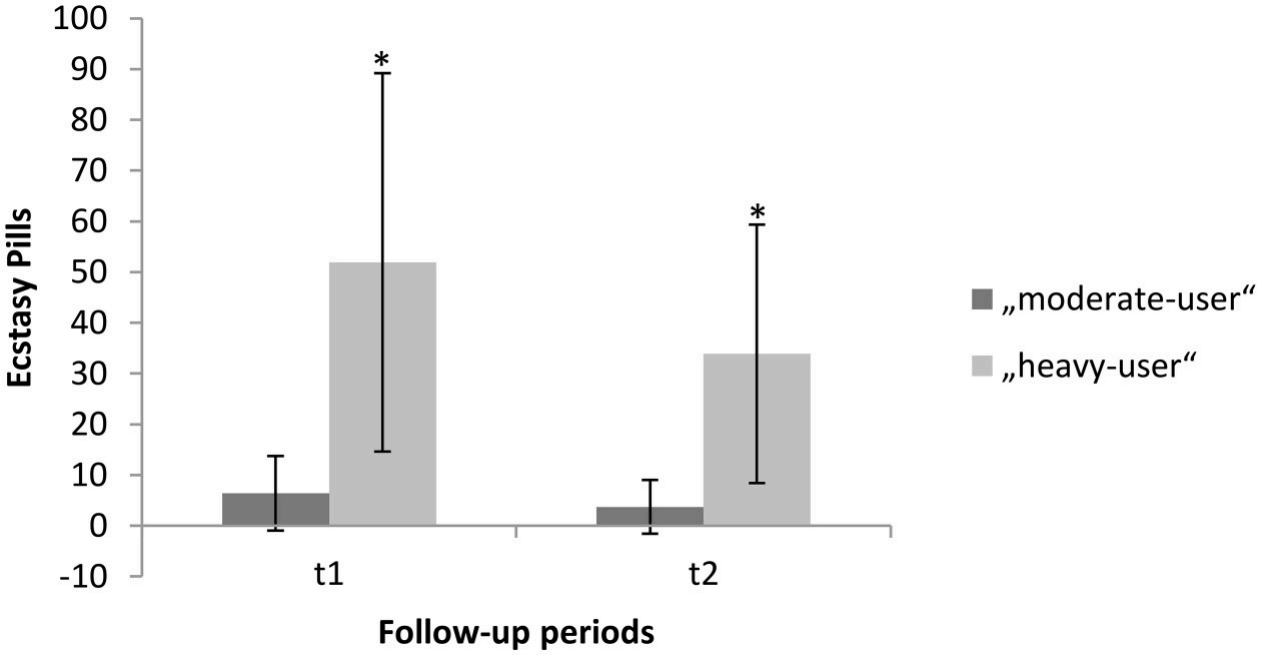
**Mean “MDMA-use” within follow-up period one and follow-up period two**. Error bars denote the standard error around the mean. ^*^Significant difference between time-point 2 (t1) and time-point 3 (t2).

A repeated measures ANOVA addressing the decline in consumption of MDMA revealed a significant interaction effect for group and time [*F*_(2, 93)_ = 0.8654, *p* = 0.000]. The corresponding within-subject contrasts showed significant changes in consumption of MDMA between time-point 2 (t1) and time-point 3 (t2). With respect to the reported means in parenthesis, these results indicate a significant decline in MDMA-use in the heavy-user group between t1 and t2 (see also Figure 1). For each group, gender distribution, mean age, mean duration of cannabis use before the baseline, mean duration of cannabis use between the baseline and the second assessment, mean days since the last cannabis use at the third assessment, Raven score and corresponding standard deviations are provided in Table 1. The groups differed regarding the mean age, use of cocaine, solvent/inhalants, and amphetamines. Therefore, these variables beside amphetamine were entered as covariates into the analysis. There was no difference in the use of hallucinogens and cannabis.

**Table 1:** **Group characteristics**.

	**Female/Male^e^**	**Age^d^**	**Cannabis use at baseline^a^^,^^d^**	**Cannabis use within follow-up^a^^,^^d^**	**Days since last cannabis use^b^^,^^d^**	**Raven score^c^^,^^d^**
Non-users	9/22	23.77 (±3.87)	46.09 (±34.79)	11.5 (±9.99)	2.0 (±7.48)	6.03(±3.80)
Moderate-users	21/34	21.87 (±3.78)	41.15 (±41.48)	16.71 (±21.78)	1.03 (±2.76)	7.78 (±6.68)
Heavy-users	3/7	26.70 (±7.54)	58.30 (±72.10)	10.80 (±14.41)	2.40 (±5.36)	11.10 (±6.15)
*F*-/*P*-value	830/660	6.08(2.93)/0.003	0.687(2.93)/0.506	1.070(2.93)/0.347	0.310(2.93)/0.735	2.92(2.93)/0.059

*Frequencies of gender, mean age, mean duration of cannabis use, mean days since last cannabis use and mean Raven score for each users group. Standard Deviations are given in parenthesis*.

a *Regular use is measured in months*.

b *Measured at third assessment*.

c *Measurement of general intelligence (lower score indicates better performance)*.

d *Computed by means of a One-way ANOVA between non-users (0 pills), moderate-users (1–49 pills), and heavy-users (50 or more pills) of 3,4-Methylenedioxymethamphetamine (MDMA)*.

e *Computed by means of chi-square test*.

As expected, a high concomitant use of amphetamines in the moderate- and heavy-user group was found (correlation MDMA and amphetamine use: *r* = 0.304, *p* = 0.003). In addition, a high concomitant use of cocaine in the moderate- and heavy-user group was also found (correlation MDMA and cocaine use: *r* = 0.210, *p* = 0.04). The corresponding means and standard deviations of the use of concomitant substances are provided in Table 2. Urine screens of all participants that were included in the analyses were free of amphetamines, benzodiazepines, cocaine, methadone, and MDMA. Moreover, the hair samples that were randomly taken and screened by, the Institute of Legal Medicine at the University of Cologne confirmed the subjects' self-reported substance use in all cases except one (which was excluded from the analyses).

**Table 2:** **Concomitant illicit substances use**.

	**Cannabis^a^**	**Cocaine^a^**	**Hallucinogens^b^**	**Solvents/Inhalants^b^**	**Amphetamines^a^**
Non-users	39.77 (±53.67)	0.53 (±2.08)	0.0 (±0.0)	23.77 (±3.87)	3.24 (±7.68)
Moderate-users	74.17 (±130.69)	9.55 (±55.62)	0.50 (±2.57)	21.87 (±3.78)	19.59 (±28.49)
Heavy-users	72.34 (±78.23)	110.58 (±341.33)	0.50(±1.58)	26.70 (±7.54)	110.67 (±341.33)
*F*-/*P*-value	1.13 (2/93)/0.327	12.08 (2.93)/0.026	0.641(2.93)/0.529	4.62(2.93)/0.012	12.08(2.93)/0.000

*Mean cannabis, cocaine, hallucinogens, solvents/inhalants, amphetamines use for each user group between the baseline assessment and the third assessment. Standard deviations are given in parenthesis*.

a *Cumulative use measured in grams*.

b *Cumulative use measured in occasions from the baseline (t0) until the second assessment (t2)*.

### Health behavior

A one-way analysis of variance was conducted with respect to the health behavior of the participants. There was no significant difference between the groups regarding any of the variables. The means, standard deviations, and significance levels are presented in Table 3.

**Table 3:** **Health behavior**.

	Satisfaction with diet^*^	Exercise^a^^**^	Alcohol use^b^^**^	Medical treatment^c^^**^	**Sleep^d^**	Subjective well-being^e^^*^
Non-users	2.12 (±4.84)	15.51 (±2.15)	7.54 (1.52)	8.35 (±1.62)	6.45 (±2.21)	1.39 (±1.30)
Moderate-users	2.72 (±5.02)	14.87 (±3.29)	7.54 (1.75)	8.52 (±1.76)	7.18 (±2.68)	1.45 (±1.18)
Heavy-users	3.30 (±5.61)	14.09 (2.42)	8.00 (1.41)	9.40 (±3.33)	8.40 (±4.03)	1.20 (±1.81)
*F*-/*P*-value	0.251 (2.93)/0.818	0.515(2.92)/0.599	0.338(2.93)/0.976	1.12(2.93)/0.328	2.11(2.92)/0.127	0.168(2.93)/0.846

*Means of the follow-up period between the first assessment and the second assessment. Standard Deviations are given in parenthesis. Computed by means of a One-way ANOVA between non-users (0 pills), moderate-users (1–49 pills), and heavy-users (50 or more pills) of 3,4-Methylenedioxymethamphetamine (MDMA)*.

a *Frequency of exercise*.

b *Mean consumption of alcoholic drinks*.

c *Frequency of intake of different drugs*.

d *Hours per night*.

e *General satisfaction with one's life*.

* *Measured by means of a 7-point likert scale (−3 = very dissatisfied; 3 = very satisfied)*.

** *Measured by means of a four-point likert scale (1 = never; 4 = daily) by adding all items scores*.

### Performance effects

This was a mixed ANOVA with group (three levels) as between-subject factor and time (three levels) as within-subject factor.

The repeated measures ANOVA addressing attention and information processing speed revealed no significant interaction effect for group and time [*F*_(12, 168.00)_ = 0.823, *p* = 0.627]. Mean test scores for each group as well as significance levels of the corresponding tests of univariate test effects are provided in Table 4.

**Table 4:** **Mean Neuropsychological Test Score for 3,4-methylenedioxymethamphetamine (MDMA) users and non-users related to attention and information processing speed**.

**Test**	**Non-users**			**Moderate-users**			**Heavy-users**			**Sig**	**η^2^**
	**Mean (*SD*) (*n* = 31)**			**Mean (*SD*) (*n* = 55)**			**Mean (*SD*) (*n* = 10)**				
	**B**	**AS 1**	**AS 2**	**B**	**AS 1**	**AS 2**	**B**	**AS 1**	**AS 2**		
TMT-A	25.76 (7.9)	23.30 (6.9)	21.17 (8.9)	27.61 (9.45)	22.60 (8.47)	20.31 (5.26)	25.03 (5.0)	25.39 (6.07)	22.58 (4.5)	0.256	0.029
ST-A	29.00 (7.5)	26.62 (4.7)	25.55 (3.2)	29.89 (8.77)	26.85 (4.20)	26.49 (4.58)	29.00 (4.4)	30.84 (12.0)	25.95 (2.9)	0.908	0.003
ST-B	44.33 (8.2)	42.65 (7.2)	41.46 (6.4)	45.44 (11.2)	42.11 (6.93)	40.31 (8.14)	48.20 (7.4)	46.57 (6.57)	45.00 (4.5)	0.739	0.009
DST	61.84 (8.8)	67.19 (9.5)	69.13 (8.8)	61.69 (11.6)	65.58 (11.8)	68.67 (12.0)	57.10 (9.5)	60.10 (10.1)	61.60 (9.5)	0.761	0.010

*B, baseline measurement; AS, assessment; TMT-A, Trail-Making Test A; ST-A, Stroop Task A reading condition; ST-B, Stroop Task B color naming condition; DST, Digit Symbol Test*.

The repeated measures ANOVA addressing episodic memory revealed a marginal significant interaction effect for group and time [*F*_(32, 148.00)_ = 1.432, *p* = 0.080, η^2^ = 0.236]. Significant interaction between group and time were found regarding univariate tests for the immediate recall test [*F*_(4, 175.30)_ = 3.939, *p* = 0.026, η^2^ = 0.060] and the delayed recall test [*F*_(4, 177.883)_ = 3.996, *p* = 0.028, η^2^ = 0.059].

**Figure 2:**
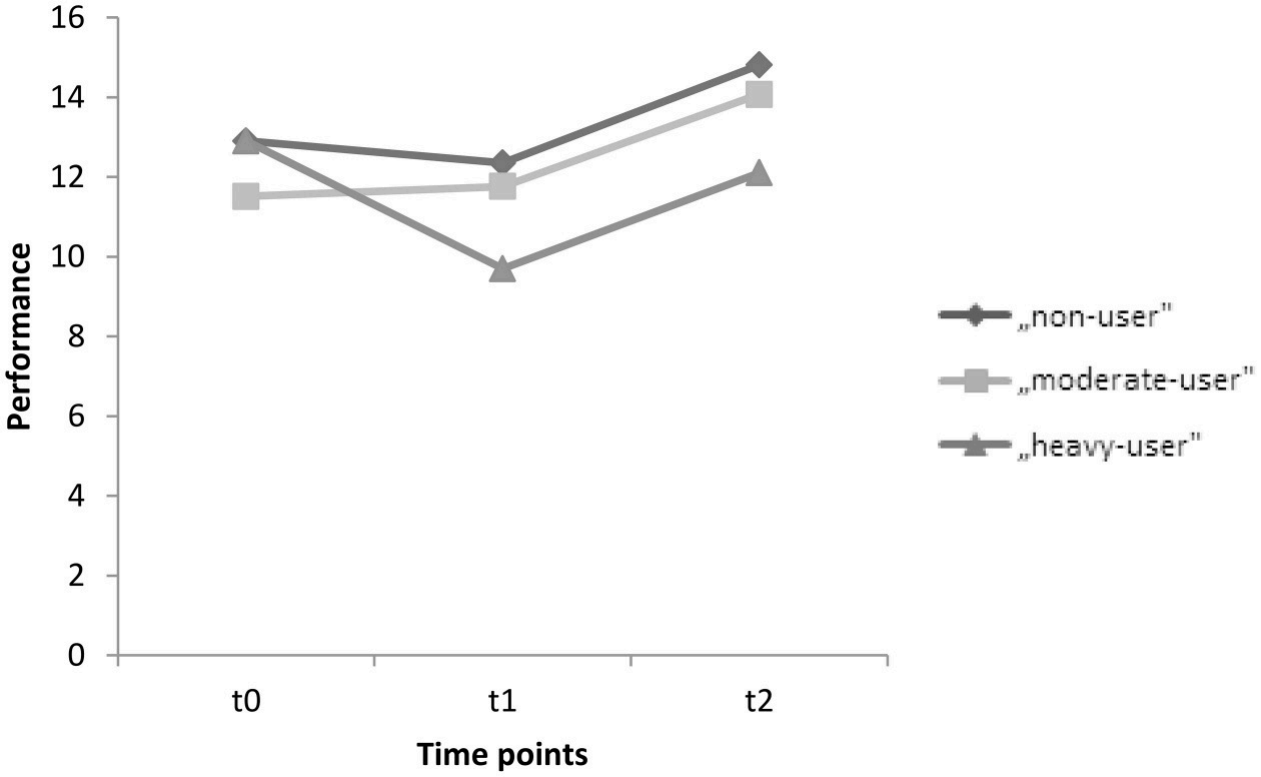
**Mean performances on the immediate recall test for time points t0, t1, and t2 for the groups, non-users, moderate-users, and heavy-users**.

The corresponding within-subject contrasts showed significant changes in performance between time point 1 (t0) and time point 2 (t1) [*F*_(2, 89)_ = 4.323, *p* = 0.016], and no significant changes between time point 2 (t1)and time point 3 (t2) [*F*_(2, 89)_ = 0.021, *p* = 0.979] regarding the immediate recall test. With respect to the reported means in Table 5, these results indicate an increase in performance in immediate recall for the non-user and moderate-user groups, with a decline in performance in the heavy-user group between t0 and t1. However, the mean test scores indicate an increase in performance in all groups between t1 and t2. Scores on the immediate recall test for each group between t0 and t2 are also represented graphically in Figure 2.

**Table 5:** **Mean Neuropsychological Test Score for 3,4-methylenedioxymethamphetamine (MDMA) users and non-users related to episodic memory**.

**Test**	**Non-users**			**Moderate-users**			**Heavy-users**			Sig.^*^	**η^2^**
	**Mean (*SD*) (*n* = 31)**			**Mean (*SD*) (*n* = 55)**			**Mean (*SD*) (*n* = 10)**				
	**B**	**AS 1**	**AS 2**	**B**	**AS 1**	**AS 2**	**B**	**AS 1**	**AS 2**		
RAVLT A^a^	7.19 (2.02)	7.39 (2.39)	7.61 (2.06)	7.18 (2.00)	7.07 (1.87)	7.47 (2.19)	7.00 (2.36)	6.60 (1.17)	6.40 (1.43)	0.934	0.005
RAVLT B^b^	6.35 (2.12)	6.42 (2.03)	6.16 (1.79)	6.36 (2.23)	6.22 (1.93)	6.24 (2.26)	5.10 (2.24)	5.20 (1.93)	5.60 (2.68)	0.967	0.003
RAVLT C^c^	12.32 (2.18)	11.94 (2.62)	12.13 (2.32)	11.96 (2.42)	12.15 (2.77)	11.55 (2.96)	10.80 (2.35)	9.70 (3.09)	10.20 (3.43)	0.288	0.028
RAVLT D^d^	1.23 (1.63)	1.84 (2.18)	1.55 (1.50)	1.42 (2.00)	1.13 (1.65)	2.38 (2.71)	1.30 (1.95)	2.38 (2.71)	2.00 (1.41)	0.136	0.039
RAVLT E^e^	14.19 (1.78)	14.26 (0.97)	14.42 (1.03)	13.98 (1.39)	13.67 (1.65)	13.87 (1.55)	13.20 (1.87)	13.00 (1.94)	11.70 (3.20)	0.106	0.042
RAVLT F^f^	4.39 (0.99)	4.06 (1.24)	3.97 (1.34)	4.33 (1.04)	4.22 (1.08)	4.22 (1.07)	4.70 (0.68)	4.60 (0.84)	4.60 (0.84)	0.773	0.010
LGT 3 logos A^g^	12.90 (2.80)	12.35 (2.47)	14.81 (2.98)	11.51 (3.48)	11.76 (3.33)	14.07 (3.91)	12.90 (3.90)	9.70 (1.95)	12.10 (2.69)	**0.026**	0.060
LGT 3 logos B^h^	11.58 (3.20)	12.00 (2.86)	13.87 (2.93)	10.84 (3.56)	11.13 (3.75)	13.35 (4.15)	12.40 (3.47)	10.00 (2.71)	10.50 (3.54)	**0.028**	0.059

B, baseline measurement; AS, assessment; SD, standard deviation. P-values refer to tests of univariate test effects of the corresponding repeated measures ANOVA. Significant

* *P-values are given in bold*.

a *Rey Auditory Verbal Learning Test (RAVLT) A: immediate recall*.

b *RAVLT B: total acquisition*.

c *RAVLT C: recall after interference*.

d *RAVLT D: loss after interference*.

e *RAVLT E: recognition performance*.

f *RAVLT F: repetitions required for learning*.

g *Lern- und Gedächtnistest (LGT) 3 A: immediate recall*.

h *LGT 3 B: delayed recall*.

**Figure 3:**
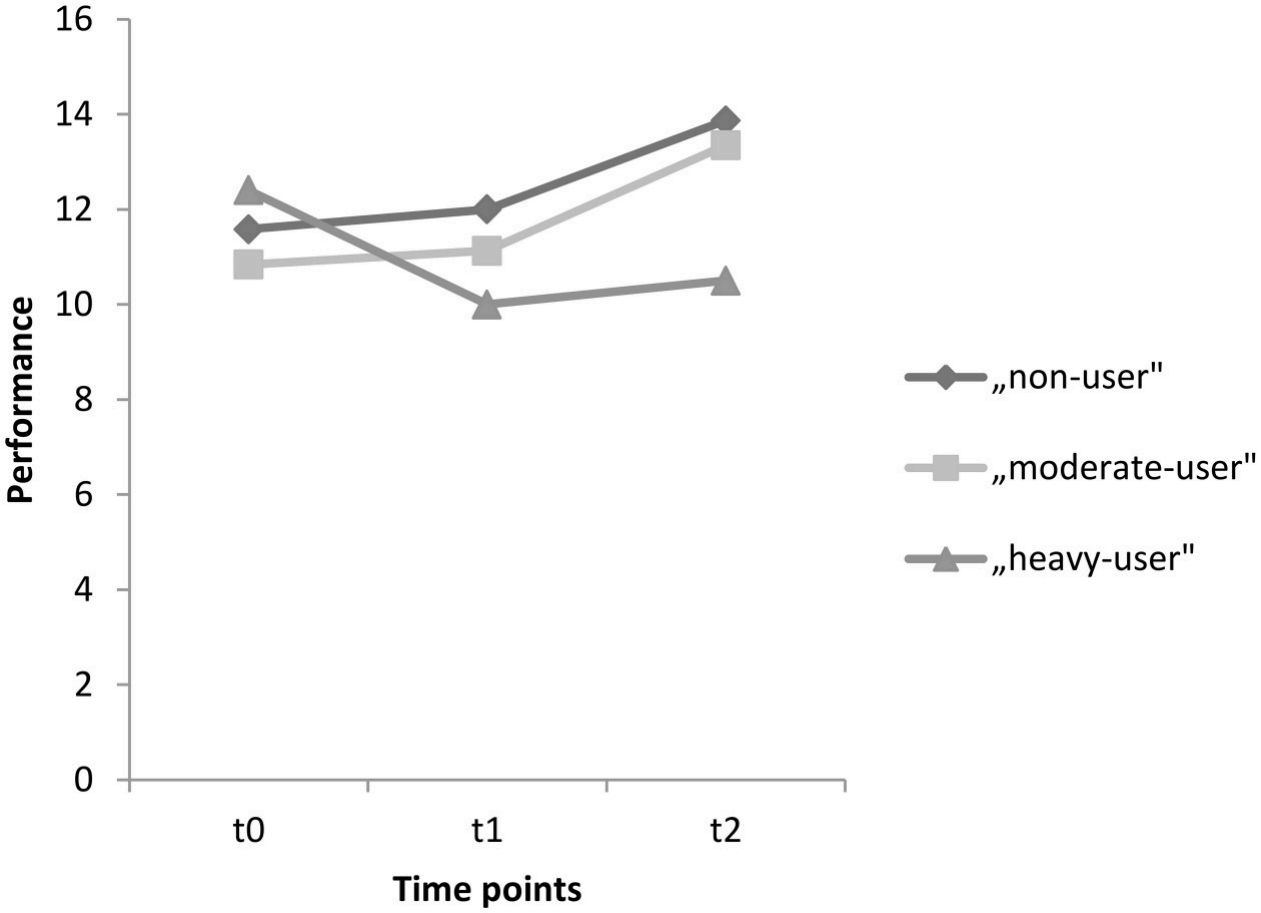
**Mean performances on the delayed recall test for time points t0, t1, and t2 for the groups, non-users, moderate-users, and heavy-users**.

For the delayed recall test, the corresponding within-subject contrasts showed significant changes in performance between time points t0 and t2 [*F*_(2, 89)_ = 5.048, *p* = 0.008]. No changes in performance were found between time points t0 and t1 [*F*_(2, 89)_ = 2.199, *p* = 0.117], nor between t1 and t2 [*F*_(2, 89)_ = 1.004, *p* = 0.370]. With respect to the reported means in Table 5, these results indicate that the non-user and the moderate-user groups increased in performance between t0 and t2, without an increase in the heavy-user group. Mean test scores for each group as well as significance levels of the corresponding univariate tests are provided in Table 5. Scores on the delayed recall test for each group between t0 and t2 are also represented graphically in Figure 3.

The last repeated measures ANOVA addressing frontal/executive functioning also did not reveal a significant interaction effectfor group and time [*F*_(12, 168.00)_ = 0.823, *p* = 0.627]. The mean test scores for each group as well as significant levels of the corresponding tests of univariate test effects are provided in Table 6. By entering amphetamines as a covariate into the repeated measure ANOVA, the effects for the LGT3-LOGO-Test do not remain marginally significant.

**Table 6:** **Mean Neuropsychological Test Score for 3,4-methylenedioxymethamphetamine (MDMA) users and non-users related to frontal/executive functioning**.

**Test**	**Non-users**			**Moderate-users**			**Heavy-users**			**Sig**.	**η^2^**
	**Mean (*SD*) (*n* = 31)**			**Mean (*SD*) (*n* = 55)**			**Mean (*SD*) (*n* = 10)**				
	**B**	**AS 1**	**AS 2**	**B**	**AS 1**	**AS 2**	**B**	**AS 1**	**AS 2**		
TMT-B	66.87 (23.8)	51.63 (18.4)	54.03 (17.5)	66.87 (26.2)	54.95 (18.6)	53.71 (17.7)	79.19 (22.8)	63.63 (25.3)	63.90 (10.9)	0.855	0.006
ST-C	73.52 (13.8)	67.78 (14.1)	66.66 (11.6)	72.95 (14.7)	66.58 (14.6)	65.28 (10.9)	83.91 (17.8)	74.28 (15.8)	71.40 (10.8)	0.981	0.002
DSB	8.19 (1.97)	7.81 (2.32)	9.81 (4.69)	7.80 (2.27)	8.49 (2.60)	8.62 (2.10)	7.10 (2.64)	7.20 (2.04)	8.00 (2.16)	0.073	0.049

*B, baseline measurement; AS, assessment; SD, standard deviation. P-values refer to the significance levels of univariate test effects of the corresponding repeated measures ANOVA. TMT-B, Trail-Making Test B; ST-C, Stroop Task C interference condition; DSB, Digit Symbol backwards Test; SD, standard deviation*.

## Discussion

The current prospective study investigated the effect of MDMA on cognitive performances in incipient MDMA users over the course of a 2-year period. The results of the first follow-up assessment showed significant deficits in MDMA users in visual paired association learning after a period of 1 year (Wagner et al., 2013). Based on those results it was hypothesized that the sustained use of MDMA over a period of 2 years would lead to further decline in cognitive performance, especially in heavy MDMA users. The same cognitive test battery which was used during the first assessment, including tests of the subject's attention and information processing speed, memory, working memory and executive functioning, were also used during the second assessment. Out of the initial 149 subjects who participated in the first assessment, 96 subjects participated in the second assessment after 2 years. The analyzed data showed no differences concerning possible confounding variables, including the subjects' cannabis use, alcohol use, and their current medical-treatment, participation in sports, diet, satisfaction with sleep, and feelings of subjective well-being. However, the groups did differ with regard to the subjects' mean age, their use of cocaine, solvents, inhalants, and amphetamines, as well as differences with regard to the subjects' general intelligence. Therefore, these variables were considered as possible confounding variables.

The repeated measures ANOVA addressing episodic memory indicated a specific effect regarding paired association learning and MDMA use. A marginally significant interaction effect of group and time for both immediate and delayed recall memory subtests from the *Lern- und Gedächtnistest (LGT; Bäumler,* *1974**)* was suggested. The analyzed data showed differences in performance among the groups between the baseline and the first assessment for the immediate recall memory test. Based on these results, it seems that the non-user and moderate-user groups increased in performance between the baseline and the first assessment. Furthermore, it appears that the performance of the heavy-user group decreased in the first follow-up period. These results are in line with the already published findings from the study of Wagner et al. (2013) that demonstrated a dose-related neurotoxic effect of MDMA on visual paired associates learning. However, it seems that with respect to the second follow-up period, this effect no longer occurred. Based on the results of the second follow-up assessment, it appears that the performance of all groups improves with regard to the immediate recall test after a period of 2 years. The findings of the current study for the first follow-up period are congruent with previous results from other investigations that found deficits in associative learning with poorer immediate and delayed recall abilities in MDMA users compared to non-users (Daumann et al., 2005; Montgomery et al., 2005; Quednow et al., 2006; Parrott, 2013a). However, as already mentioned, the analyzed data showed a similar improvement in performance for the immediate recall test for all groups between the first assessment and the second assessment. These results are consistent with the findings of de Sola Llopis et al. (2008). In their study, they also found an improvement in the performance of MDMA users after a follow-up period of 2 years. However, the overall performance of the MDMA users was still lower when compared to the non-users. They postulated that the subjects' improvement in performance could be due to practice effects through their repeated exposure to the test material. Therefore, it may also be possible that the participants in the current study performed better because they were better acquainted with the test material. The use of parallel versions from neurocognitive tests could be a solution to prevent such practice effects in future studies. Perhaps another explanation for the improvement in performance in the heavy-user group could be a decrease in their MDMA use between the first and second assessments (see Figure 1). Similar results were found in other longitudinal studies: Heavy-users improved their performance on an immediate recall test in which the average use of MDMA had decreased between the first and second assessments (Thomasius et al., 2003; Gouzoulis-Mayfrank et al., 2005). A last explanation for the marginal significant effect of MDMA-use between the groups could be that the non-user group also could have been impaired in cognitive performance before participating in this study due to their little experience with MDMA. Because even a small cumulative dose of MDMA, is associated with a decline in cognitive performance (Schilt et al., 2007). For the future, it would be interesting to add a group without any drug experience to rule out preexisting cognitive impairments due to MDMA-use in the control-group.

With respect to performance on the delayed recall test, it seems that the groups changed in performance between the baseline assessment and the second assessment. It appears that an improvement in performance occurred among the non-users and moderate-users, but not in the heavy-user group. More precisely, a performance decline in heavy-users took place between baseline and the first assessment, with no further deterioration in performance between the first and the second assessments. This could indicate a dose-related neurotoxic effect of MDMA within the first follow-up period—as was already found in the study of Wagner et al. (2013)—but not for the second-follow-up period. Therefore, it seems that continuing MDMA, use did not lead to further decrease in performance on the delayed recall test after 2 years, a result which corresponds with the previous findings of Gouzoulis-Mayfrank et al. (2005). Furthermore, no significant changes in performance within the 2-year follow-up period were found, results which are similar to the findings of Brown et al. (2010). Brown and colleagues also established significant effects on association learning tasks with regard to MDMA users and non-users, but no differences concerning other memory tasks. However, it has not been established whether these other measures of cognitive performance may also decrease over time with prolonged use of MDMA over the subject's lifetime. Therefore, longitudinal studies including follow-up assessments over a period of several years are needed.

As already established in our analysis of the data from the first follow-up period (Wagner et al., 2013), a significant intercorrelation was found between the subjects' use of MDMA and amphetamine in the second follow-up period. It still remains unclear whether or not the impairments in visual pair associates learning can be ascribed to the subjects' use of MDMA alone or whether it is also due to their concomitant use of amphetamine. However, in their study, Gouzoulis-Mayfrank et al. (2003) attributed the subjects' performance on the same immediate and delayed recall test to their use of MDMA rather than to their polydrug-use of MDMA and amphetamine. More research concerning memory deficits in humans with respect to the neurotoxic effects of amphetamine are needed. Furthermore, there was also a significant intercorrelation between the subjects' use of MDMA and cocaine. However, the confounding effect of cocaine did not fully account for the differences between the groups on the visual paired association task. Even after excluding cocaine as a covariate, the results on the visual paired association task remained marginal significant. Even though dose-related neurotoxic effects of MDMA have been established, among incipient MDMA users in their first year of regular use, it seems that this effect no longer exists or at least diminishes after a second year of MDMA use. In this respect, the findings of the current study could be interpreted as contradicting the MDMA-related memory decline in continuing users. Otherwise, a further decrease in performance within the second year, would be expected. Another explanation could be that the neurotoxic potential of MDMA displays its effect on memory at the beginning of regular MDMA use in novice users without an exponential deterioration through continued use. In a recent study, Taurah et al. (2013), argued that prolonged use of MDMA is a weak predictor for memory deficits. Instead, they argue that it is a combination of MDMA use with other drugs that seems crucial for memory deficits in recent and past MDMA users (Taurah et al., 2013). However, most studies reveal that prolonged lifetime use of MDMA leads to memory deficits in MDMA users (Parrott et al., 2002; Parrott, 2013a). Moreover, the performance of MDMA users gets worse when they are confronted with supraspan tasks, where their memory systems become overloaded beyond their normal capacities (Parrott, 2013a). In this respect, the LGT 3 is such a supraspan task (Bäumler, 1974). This test has been shown to be sensitive in detecting performance deficits in MDMA users (Gouzoulis-Mayfrank et al., 2003).

The recent literature shows that impairment in MDMA users can be found in almost all cognitive domains (Parrott, 2013b). In their study, Quednow et al. (2006) found deficits in verbal declarative memory in MDMA users compared to non-users. The subjects' verbal declarative memory was assessed with the Rey Auditory Verbal Learning Test (RAVLT; Heubrock, 1992), which was likewise used in our current study as an assessment tool. However, as compared to our study, Quednow et al. (2006) looked at a group of former MDMA users with a longer lifetime and higher cumulative use of MDMA. Regarding to the longer lifetime use and higher cumulative use of MDMA, it might be possible that the performance of MDMA users on the declarative memory task in the current study may continue to decline if they continue to use MDMA in the following years.

The findings on the tests of executive functioning are consistent with the previous study of Halpern et al. (2011). They also found no differences on tests of executive functioning (Trail-Making Test A and B), except with the digit span test backwards from the WAIS 3. Although MDMA users showed impairments on the digit span test backwards, significant differences were only established between the non-user and moderate-user groups, and not between the heavy-user and non-user groups. This is inconsistent with the dose-related neurotoxic effect hypothesis of MDMA. Moreover, no differences between the groups were found in performance on the Stroop Tasks, a result which corresponds with our findings. In summary, the results of the current study indicate a MDMA-related performance decrease in heavy MDMA users on a visual paired association task. A reason for the performance decrease in heavy MDMA users on visual paired association tasks could be hippocampal dysfunction, because the hippocampus plays a crucial role in learning processes and memory, and it is particularly vulnerable to the neurotoxic effects of MDMA (Hatzidimitriou et al., 1999; Daumann et al., 2005; Brown et al., 2010; Pergola and Suchan, 2013). Interestingly, in a recent prospective fMRI study by Becker et al. (2013), continuing MDMA users showed, in contrast to abstinent MDMA users, a decrease in encoding-related activity in the left parahippocampus during an association memory task. However, no differences in performance on this task were found between the groups. This suggests that MDMA users are still able to perform in a normal range despite an MDMA-specific dysfunction of the parahippocampus. One possible explanation could be that MDMA users do not want to believe that their results on memory performance tests could be negatively affected by their MDMA use. Consequently, they may try harder to reach normal results and thereby compensate for the dysfunction of their parahippocampus. In a recent functional near-infrared spectroscopy (fNIRS) study by Roberts, Wetherell, Fisk and Montgomery (Roberts et al., 2015) they found also no behavioral performance differences on a high mental workload task between MDMA-users and non-users. However, in comparison to non-users, MDMA-users showed an aberrant neuronal functioning in the dorsolateral prefrontal cortex (DLPFC), suggesting that MDMA-users have neurological impairments in these networks. Also in Electroencephalography (EEG) studies, neuronal changes in MDMA-users are found in the absence of a decrease in behavioral performances (Burgess et al., 2011; Roberts et al., 2013). Therefore, it seems that performance on cognitive tasks between MDMA-users and non-users alone are not sensitive enough to measure cognitive impairments induced by MDMA-use.

Despite the empirical evidence found in animal studies for the neurotoxic effect of MDMA in the hippocampus and other brain regions, the underlying molecular mechanisms have not been fully clarified. There are different factors that seem to contribute to the neurotoxic effect of MDMA. For example, heightened ambient temperature influences the metabolites of MDMA which are associated with the induction of 5-HT toxicity (Moon et al., 2008; Capela et al., 2009) and decreases the activity of tryptophan hydroxylase (TPH), an enzyme that is required for the synthesis of 5-HT (Moon et al., 2008). Oxidative stress induced through MDMA administration leads to an increase in reactive oxygen species (ROS) and decreases the antioxidant defense of cells, which leads to cell damage and mitochondrial dysfunction (Moon et al., 2008; Capela et al., 2009). As a consequence of an increased monoamine oxidase metabolism of monoamine neurotransmitters (dopamine, noradrenaline, adrenaline, and serotonin), more toxic metabolites can be found in cells. This subsequently leads to cell damage.

However, the underlying mechanism of MDMA, which can cause neurodegeneration and changes in the serotonin system, is still difficult to identify in humans (Green et al., 2012). Most evidence regarding the underlying molecular mechanisms came from studies using laboratory species. This creates difficulties in translating such information to human MDMA users (Green et al., 2012). Furthermore, there are findings which are inconsistent with the neurodegeneration hypothesis. For example, serotonergic fibers have been found to remain in rats even after MDMA administration and indeed to recover from the neurotoxic effects of MDMA (Hatzidimitriou et al., 1999; Kish et al., 2010; Biezonski and Meyer, 2011). With respect to the empirical evidence of the neurodegenerating effects of MDMA on human users, it is therefore important to consider that serotonergic deficits are not necessarily the result of axonal damage. Therefore, Biezonski and Meyer (2011) noted that “MDMA can certainly be considered as neurotoxic in terms of causing serotonergic dysfunction.”

It should be noted that the current study is not free of certain methodological limitations, as we have already discussed in our previous paper. Like other open-trial studies, this study was not experimental, and, therefore, it provides no causal interpretation of the effect of MDMA on brain regions which are associated with specific cognitive performance. This study began with a large sample of 149 subjects. However, only 96 subjects remained for the second follow-up assessment, and most of these were non-users or moderate-users. Therefore, the subjects were unevenly distributed over the groups, with a small sample size of 10 subjects in the heavy-user group. In addition, the remaining 10 participants in the heavy-users group might be actually highly motivated and that might constitute a bias in terms of performance and might partially explain the results found in the present study. Furthermore, in the recent study MDMA-users consumed cannabis almost twice as much as non-users. Even though the concomitant use of cannabis did not differ significantly between the groups, a possible confound of cannabis cannot be completely ruled out. Some investigators have suggested a possible neuroprotective effect of Tetrahydrocannabinol (THC) against MDMA neurotoxicity in laboratory animals (Tourino et al., 2010; Lopez-Rodriguez et al., 2014). In this regard, cannabis might be preventing a performance decline in moderate-users but not in heavy-users due to their excessive use of MDMA. In a fMRI-study of Daumann et al. (2003) pure MDMA-users presented lower activations of different brain regions during a working-memory task in comparison to polyvalent- and non-users, suggesting a neuroprotective effect of cannabis against MDMA neurotoxicity.

Furthermore, in the recent study a significant difference between the groups in their use of cocaine was found. Although, no interaction effect of MDMA and cocaine was found on cognitive functioning, a confounding effect of cocaine cannot completely ruled out, too. Investigators found cognitive impairments on different cognitive domains in cocaine-users (Potvin et al., 2014). Thus, in the recent study, the impairments in visual pair associates learning cannot only be attributed to MDMA alone but also to the concomitant use of cocaine and cannabis, which is a common limitation to prospective MDMA-studies. Further limitations, which are common to all longitudinal, MDMA research in humans have already been intensively discussed. For these, we refer the reader to our already published study (Wagner et al., 2013).

In conclusion, the already published findings of the first follow-up period seem to be consistent with the findings of the current study. In this study, we found an MDMA-related decline in memory performance on a visual paired association test in the first follow-up period in heavy MDMA users, suggesting an MDMA-related dysfunction of the hippocampal regions. However, no further deterioration was observed in the second follow-up period, that could be due to methodological limitations of the recent study, which are inherent to open-trial studies and mentioned below.

### Conflict of interest statement

The authors declare that, except for income received from their primary employer, no financial support or compensation has been received from any individual or corporate entity over the past 36 months for research or professional service, and that there are no personal financial holdings that could be perceived as constituting a potential conflict of interest.

## Footnotes

^1^UNODC, World Drug Report 2013 (United Nations publication, Sales No. E.13.XI.6).

^2^UNODC, World Drug Report 2012 (United Nations publication, Sales No. E.12.XI.1).

## References

[B1] American Psychiatric Association (1994). Diagnostic and Statistical Manual of Mental Disorders, 4th Edn Washington, DC: American Psychiatric Association.

[B2] Arias-Cavieres A. Rozas C. Reyes-Parada M. Barrera N. Pancetti F. Loyola S.. (2010). MDMA (“ecstasy”) impairs learning in the Morris Water Maze and reduces hippocampal LTP in young rats. Neurosci. Lett. 469, 375–379. 10.1016/j.neulet.2009.12.031 20026184

[B3] Bäumler G. (1974). Lern- und Gedächtnistest LGT-3, Hogrefe, Göttingen.

[B4] Bäumler G. (1985). Farb-Wort-Interferenztest (FWIT) nach J. R. Stroop. Göttingen: Hogrefe.

[B5] Becker B. Wagner D. Koester P. Bender K. Kabbasch C. Gouzoulis-Mayfrank E.. (2013). Memory-related hippocampal functioning in ecstasy and amphetamine users: a prospective fMRI study. Psychopharmacology (Berl) 225, 923–934. 10.1007/s00213-012-2873-z 23001254

[B6] Biezonski D. K. Meyer J. S. (2011). The Nature of 3, 4-Methylenedioxymethamphetamine (MDMA)-Induced Serotonergic Dysfunction: Evidence for and Against the Neurodegeneration Hypothesis. Curr. Neuropharmacol. 9, 84–90. 10.2174/157015911795017146 21886568 PMC3137208

[B7] Bolla K. I. McCann U. D. Ricaurte G. A. (1998). Memory impairment in abstinent MDMA (“Ecstasy”) users. Neurology 51, 1532–1537. 10.1212/WNL.51.6.1532 9855498

[B8] Brown J. McKone E. Ward J. (2010). Deficits of long-term memory in ecstasy users are related to cognitive complexity of the task. Psychopharmacology (Berl) 209, 51–67. 10.1007/s00213-009-1766-2 20119830

[B9] Burgess A. P. Venables L. Jones H. Edwards R. Parrott A. C. (2011). Event related potential (ERP) evidence for selective impairment of verbal recollection in abstinent recreational methylenedioxymethamphetamine (“Ecstasy”)/polydrug users. Psychopharmacology (Berl) 216, 545–556. 10.1007/s00213-011-2249-9 21390504

[B10] Camarasa J. Marimón J. M. Rodrigo T. Escubedo E. Pubill D. (2008). Memantine prevents the cognitive impairment induced by 3,4-methylenedioxymethamphetamine in rats. Eur. J. Pharmacol. 589, 132–139. 10.1016/j.ejphar.2008.05.014 18582864

[B11] Capela J. P. Carmo H. Remião F. Bastos M. L. Meisel A. Carvalho F. (2009). Molecular and cellular mechanisms of ecstasy-induced neurotoxicity: an overview. Mol. Neurobiol. 39, 210–271. 10.1007/s12035-009-8064-1 19373443

[B12] Daumann J. Fischermann T. Heekeren K. Henke K. Thron A. Gouzoulis-Mayfrank E. (2005). Memory-related hippocampal dysfunction in poly-drug ecstasy (3,4-methylenedioxymethamphetamine) users. Psychopharmacology (Berl) 180, 607–611. 10.1007/s00213-004-2002-8 15372137

[B13] Daumann J. Schnitker R. Weidemann J. Schnell K. Thron A. Gouzoulis-Mayfrank E. (2003). Neural correlates of working memory in pure and polyvalent ecstasy (MDMA) users. Neuroreport 14, 1983–1987. 10.1097/00001756-200310270-00021 14561934

[B14] de Sola Llopis S. Miguelez-Pan M. Pena-Casanova J. Poudevida S. Farré M. Pacifici R.. (2008). Cognitive performance in recreational ecstasy polydrug users: A two-year follow-up study. Psychopharmacol. Bull. 22, 498–510. 10.1177/0269881107081545 18208910

[B15] Dlugosch G. E. Krieger W. (1995). Der Fragebogen zur Erfassung des Gesundheitsverhaltens (FEG), Frankfurt; Swets Test Gesellschaft.

[B16] Fox H. C. Parrott A. C. Turner J. J. (2001). Ecstasy use: cognitive deficits related to dosage rather than self-reported problematic use of the drug. J. Psychopharmacol. 15, 273–281. 10.1177/026988110101500406 11769821

[B17] Gouzoulis-Mayfrank E. Fischermann T. Rezk M. Thimm B. Hensen G. Daumann J. (2005). Memory performance in polyvalent MDMA (ecstasy) users who continue or discontinue MDMA use. Drug Alcohol Depend. 78, 317–323. 10.1016/j.drugalcdep.2004.12.002 15893163

[B18] Gouzoulis-Mayfrank E. Thimm B. Rezk M. Hensen G. Daumann J. (2003). Memory impairment suggests hippocampal dysfunction in abstinent ecstasy users. Prog. Neuropsychopharmacol. Biol. Psychiatry 27, 819–827. 10.1016/S0278-5846(03)00114-3 12921915

[B19] Green A. R. King M. V. Shortall S. E. Fone K. C. (2012). Lost in translation: preclinical studies on 3,4-methylenedioxymethamphetamine provide information on mechanisms of action, but do not allow accurate prediction of adverse events in humans. Br. J. Pharmacol. 166, 1523–1536. 10.1111/j.1476-5381.2011.01819.x 22188379 PMC3419898

[B20] Halpern J. H. Sherwood A. R. Hudson J. I. Gruber S. Kozin D. Pope H. G. Jr. (2011). Residual neurocognitive features of long-term ecstasy users with minimal exposure to other drugs. Addiction 106, 777–786. 10.1111/j.1360-0443.2010.03252.x 21205042 PMC3053129

[B21] Hatzidimitriou G. McCann U. D. Ricaurte G. A. (1999). Altered serotonin innervation patterns in the forebrain of monkeys treated with (+∕−)3,4-methylenedioxymethamphetamine seven years previously: factors influencing abnormal recovery. J. Neurosci. 19, 5096–5107. 10366642 10.1523/JNEUROSCI.19-12-05096.1999 PMC6782677

[B22] Heubrock D. (1992). Der Auditiv-Verbale Lerntest (AVLT) in der klinischen und experimentellen Neuropsychologie. Z. Diff. Diagn. Psychol. 13, 161–174.

[B23] Kay C. Harper D. N. Hunt M. (2010). Differential effects of MDMA and scopolamine on working versus reference memory in the radial arm maze task. Neurobiol. Learn. Mem. 93, 151–156. 10.1016/j.nlm.2009.09.005 19766200

[B24] Kish S. J. Lerch J. Furukawa Y. Tong J. McCluskey T. Wilkins D. (2010). Decreased cerebral cortical serotonin transporter binding in ecstasy users: a positron emission tomography/[(11)C]DASB and structural brain imaging study. Brain 133, 1779–1797. 10.1093/brain/awq103 20483717 PMC2912692

[B25] Lopez-Rodriguez A. B. Llorente-Berzal A. Garcia-Segura L. M. Viveros M. P. (2014). Sex-dependent long-term effects of adolescent exposure to THC and/or MDMA on neuroinflammation and serotoninergic and cannabinoid systems in rats. Br. J. Pharmacol. 171, 1435–1447. 10.1111/bph.12519 24236988 PMC3954483

[B26] McAleer L. M. Schallert T. Duvauchelle C. L. (2013). Weekend Ecstasy use disrupts memory in rats. Neurosci. Lett. 549, 173–176. 10.1016/j.neulet.2013.05.030 23707649 PMC3729727

[B27] Montgomery C. Fisk J. E. Newcombe R. (2005). The nature of ecstasy-group related deficits in associative learning. Psychopharmacology (Berl). 180, 141–149. 10.1007/s00213-004-2131-0 15668817

[B28] Moon K. H. Upreti V. V. Yu L. R. Lee I. J. Ye X. Eddington N. D.. (2008). Mechanism of 3,4-methylenedioxymethamphetamine (MDMA, ecstasy)-mediated mitochondrial dysfunction in rat liver. Proteomics 8, 3906–3918. 10.1002/pmic.200800215 18780394 PMC2590641

[B29] Parrott A. C. (2013a). Human psychobiology of MDMA or ‘Ecstasy’: an overview of 25 years of empirical research. Hum. Psychopharmacol. 28 289–307. 10.1002/hup.2318 23881877

[B30] Parrott A. C. (2013b). MDMA, serotonergic neurotoxicity, and the diverse functional deficits of recreational ‘Ecstasy’ users. Neurosci. Biobehav. Rev. 37, 1466–1484. 10.1016/j.neubiorev.2013.04.016 23660456

[B31] Parrott A. C. Buchanan T. Scholey A. B. Heffernan T. Ling J. Rodgers J. (2002). Ecstasy/MDMA attributed problems reported by novice, moderate and heavy recreational users. Hum. Psychopharmacol. 17, 309–312. 10.1002/hup.415 12404677

[B32] Parrott A. C. Lees A. Garnham N. J. Jones M. Wesnes K. (1998). Cognitive performance in recreational users of MDMA of ‘ecstasy’: evidence for memory deficits. J. Psychopharmacol. 12, 79–83. 10.1177/026988119801200110 9584971

[B33] Parrott A. C. Milani R. M. Gouzoulis-Mayfrank E. Daumann J. (2007). Cannabis and Ecstasy/MDMA (3,4-methylenedioxymethamphetamine): an analysis of their neuropsychobiological interactions in recreational users. J. Neural Transm. 114, 959–968. 10.1007/s00702-007-0715-7 17520319

[B34] Pergola G. Suchan B. (2013). Associative learning beyond the medial temporal lobe: many actors on the memory stage. Front. Behav. Neurosci. 7:162. 10.3389/fnbeh.2013.00162 24312029 PMC3832901

[B35] Potvin S. Stavro K. Rizkallah E. Pelletier J. (2014). Cocaine and cognition: a systematic quantitative review. J. Addict. Med. 8, 368–376. 10.1097/ADM.0000000000000066 25187977

[B36] Quednow B. B. Jessen F. Kuhn K. U. Maier W. Daum I. Wagner M. (2006). Memory deficits in abstinent MDMA (ecstasy) users: neuropsychological evidence of frontal dysfunction. J. Psychopharmacol. 20, 373–384. 10.1177/0269881106061200 16574711

[B37] Raven J. Raven J. C. Court J. H. (1998). Raven Manual: Standard Progressive Matrices. Oxford: Oxford Psychologists Press.

[B38] Reitan R. M. (1992). TMT, Trail Making Test A and B. South Tucson, AZ: Reitan Neuropsychology Laboratory.

[B39] Reneman L. Majoie C. B. Habraken J. B. den Heeten G. J. (2001). Effects of ecstasy (MDMA) on the brain in abstinent users: initial observations with diffusion and perfusion MR imaging. Radiology 220, 611–617. 10.1148/radiol.2202001602 11526257

[B40] Roberts C. A. Fairclough S. H. Fisk J. E. Tames F. Montgomery C. (2013). ERP evidence suggests executive dysfunction in ecstasy polydrug users. Psychopharmacology (Berl). 228, 375–388. 10.1007/s00213-013-3044-6 23532375

[B41] Roberts C. A. Wetherell M. A. Fisk J. E. Montgomery C. (2015). Differences in prefrontal blood oxygenation during an acute multitasking stressor in ecstasy polydrug users. Psychol. Med. 45, 395–406. 10.1017/S0033291714001500 25066866

[B42] Schilt T. de Win M. M. Koeter M. Jager G. Korf D. J. van den Brink W.. (2007). Cognition in novice ecstasy users with minimal exposure to other drugs: a prospective cohort study. Arch. Gen. Psychiatry 64, 728–736. 10.1001/archpsyc.64.6.728 17548754

[B43] Stroop J. R. (1935). Studies of interference in serial verbal reactions. J. Exp. Psychol. 18, 643–662. 10.1037/h0054651

[B44] Taurah L. Chandler C. Sanders G. (2013). Depression, impulsiveness, sleep, and memory in past and present polydrug users of 3,4-methylenedioxymethamphetamine (MDMA, ecstasy). Psychopharmacology (Berl). 231, 737–751. 10.1007/s00213-013-3288-1 24114426

[B45] Tewes U. (1994). HAWIE-R. Hamburg–Wechsler-Intelligenztest für Erwachsene, Revision 1991; Handbuch und Testanweisung [HAWIE-R Hamburg-Wechsler-Intelligence-Scale for Adults, Revised 1991: Manual and Instructions.]. Bern; Göttingen; Toronto; Seattle: Verlag Hans Huber.

[B46] Thomasius R. Petersen K. Buchert R. Andresen B. Zapletalova P. Wartberg L.. (2003). Mood, cognition and serotonin transporter availability in current and former ecstasy (MDMA) users. Psychopharmacology (Berl). 167, 85–96. 10.1007/s00213-002-1383-9 12632248

[B47] Touriño C. Zimmer A. Valverde O. (2010). THC prevents MDMA Neurotoxicity in Mice. PLoS ONE 5:e9143. 10.1371/journal.pone.0009143 20174577 PMC2824821

[B48] U.N.O.o.D.a.C (UNODC) (2013). World Drug Report 2013 (New York, NY).

[B49] Wagner D. Becker B. Gouzoulis-Mayfrank E. Daumann J. (2010). Interactions between specific parameters of cannabis use and verbal memory. Prog. Neuropsychopharmacol. Biol. Psychiatry 34, 871–876. 10.1016/j.pnpbp.2010.04.004 20398718

[B50] Wagner D. Becker B. Koester P. Gouzoulis-Mayfrank E. Daumann J. (2013). A prospective study of learning, memory, and executive function in new MDMA users. Addiction 108, 136–145. 10.1111/j.1360-0443.2012.03977.x 22831704

